# Limited electricity access in health facilities of sub-Saharan Africa: a systematic review of data on electricity access, sources, and reliability

**DOI:** 10.9745/GHSP-D-13-00037

**Published:** 2013-08-14

**Authors:** Heather Adair-Rohani, Karen Zukor, Sophie Bonjour, Susan Wilburn, Annette C Kuesel, Ryan Hebert, Elaine R Fletcher

**Affiliations:** a World Health Organization, Department of Public Health and Environment, Geneva, Switzerland; b WE CARE Solar, Berkeley, CA, USA

## Abstract

**Background:**

Access to electricity is critical to health care delivery and to the overarching goal of universal health coverage. Data on electricity access in health care facilities are rarely collected and have never been reported systematically in a multi-country study. We conducted a systematic review of available national data on electricity access in health care facilities in sub-Saharan Africa.

**Methods:**

We identified publicly-available data from nationally representative facility surveys through a systematic review of articles in PubMed, as well as through websites of development agencies, ministries of health, and national statistics bureaus. To be included in our analysis, data sets had to be collected in or after 2000, be nationally representative of a sub-Saharan African country, cover both public and private health facilities, and include a clear definition of electricity access.

**Results:**

We identified 13 health facility surveys from 11 sub-Saharan African countries that met our inclusion criteria. On average, 26% of health facilities in the surveyed countries reported no access to electricity. Only 28% of health care facilities, on average, had reliable electricity among the 8 countries reporting data. Among 9 countries, an average of 7% of facilities relied solely on a generator. Electricity access in health care facilities increased by 1.5% annually in Kenya between 2004 and 2010, and by 4% annually in Rwanda between 2001 and 2007.

**Conclusions:**

Energy access for health care facilities in sub-Saharan African countries varies considerably. An urgent need exists to improve the geographic coverage, quality, and frequency of data collection on energy access in health care facilities. Standardized tools should be used to collect data on all sources of power and supply reliability. The United Nations Secretary-General's “Sustainable Energy for All” initiative provides an opportunity to comprehensively monitor energy access in health care facilities. Such evidence about electricity needs and gaps would optimize use of limited resources, which can help to strengthen health systems.

## Abstract (precis)

Only 34% of hospitals have reliable electricity access in surveyed sub-Saharan African countries. However, analysis in 2 countries indicates modest improvements in electricity access over time. Ambitious plans to improve health service delivery in sub-Saharan Africa need to address this critical issue.

## BACKGROUND

From a health and development perspective, ensuring universal access to modern energy services in health facilities in developing countries is an essential requirement for improving health and well-being. However, evidence about energy access in health care facilities in developing regions is lacking. In 2012, the United Nations (UN) Secretary-General launched the “Sustainable Energy for All” (SE4All) initiative, which aims to achieve universal access to clean and modern energy sources in households and community settings by 2030.^1^ The initiative also aims to double the global rate of energy efficiency and use of renewable energy. SE4All notes that health care facilities are a special focus on its community energy access agenda; work has already begun to define measurable access targets for electricity—one of the most widely used forms of energy in health services.

The UN Secretary-General's Sustainable Energy for All initiative aims to achieve universal access to modern energy services by 2030.

In the health-sector context, a 2012 UN General Assembly resolution defined universal health coverage as a top global priority, urging governments to move toward providing all people with access to affordable, quality health care services.^2^ The World Health Organization (WHO) defines access to essential medicines and technologies as 1 of the 4 key factors to ensuring universal health coverage.^3^ Many of these “essential technologies” require electricity, and without electricity, many health care interventions simply cannot be provided.

Although there is no unified matrix of electrical devices required for all essential health care services, access to electricity is an implicit or explicit concern of recent assessments on available technologies.^4^ For instance, a recent UN interagency list of essential medical devices for reproductive health specifically denotes those devices that require access to electricity (Table 1).^5^

**Table 1.** UN Interagency List of Essential Devices for Reproductive Health Requiring Electricity

Essential Devices	First-Level Clinic	Referral-Level Facility (Non-Hospital)
Doppler	✓	✓
Scanner, ultrasound		✓
Sterilizer, steam ∼24–40 L	✓	
Sterilizer, steam ∼39–100 L		✓
Vacuum extractor^a^		✓
Breast pump^a^		✓
**Anesthesia/Resuscitation Equipment**		
Free-standing anesthesia system		✓
Newborn incubator		✓
Patient monitor		✓
Nebulizer, atomizer, with electric compressor		✓
Phototherapy unit		✓
Pulse oximeter portable unit		✓
Resuscitation table (newborn)		✓
Resuscitation ventilator (adult/child)		✓
Electric baby warmer	✓	

Facility appliances, such as electric lights, communication equipment, water pumps, and refrigeration, are not included in the table.

a Manual version sometimes available.

Source: Adapted from the “Interagency List of Essential Medical Devices for Reproductive Health.”^5^

Initiatives to expand capacity in developing countries to prevent and treat noncommunicable diseases also are placing increased emphasis on the essential devices required, such as electrocardiograms and mammograms, most of which require significant electricity supply capacity.^6^ Immunization policy also faces an energy challenge: WHO has projected that vaccine refrigeration capacity needs to expand 8- to 10-fold by 2025 to meet the vaccine needs of a growing global population.^7^

Many essential devices used in health care services require significant electricity supply.

Even when not an outright barrier to services, presence of electricity can improve the range of potential primary care interventions.

Anecdotal evidence and findings from a country assessment^8^ indicate that, in developing countries, electricity access in health care facilities is partial and unreliable. However, trends and patterns have not been compared systematically across countries or in regions.

Establishing electricity access profiles of health care facilities in developing countries can identify settings where lack of electricity may be a severe and underreported barrier to effective health care delivery. Better data can inform innovations in the health and energy sectors, as well as direct investments in areas with greatest need. They also can document progress in closing energy gaps that may create a “silent barrier” to accessing health services, particularly for poor and vulnerable populations. Such benchmarking and monitoring is relevant not only to universal health coverage but also to better integration of health-sector issues into sustainable development goals and targets. This systematic review and analysis aims to expand our knowledge and understanding of the state of electricity access in health facilities of developing countries.

## METHODS

### Data Sources and Search Strategy

We reviewed available data on electricity access from websites dedicated to assessing health facility equipment, including:

- Service Provision Assessment (SPA) implemented under the MEASURE Demographic and Health Survey program, supported by the U.S. Agency for International Development (USAID)^9^
- A similar WHO tool, called Service Availability Mapping (SAM)^10^
- A more recent tool, the Service Availability and Readiness Assessment (SARA), developed jointly between WHO and USAID, which aims to harmonize diverse approaches to facility assessment^11^

We also searched the websites of the International Health Facility Assessment Network (IHFAN) and the Global Fund to Fight AIDS, Tuberculosis and Malaria (for their Technical Evaluation surveys).^12^-^13^ From these websites, we identified a cluster of nationally representative facility data sets from sub-Saharan Africa. Outside sub-Saharan Africa, we found nationally representative data sets for only 3 developing countries (Bangladesh, Egypt, and Guyana).

As a result, we narrowed our systematic literature review to sub-Saharan African countries and searched PubMed using the following search terms: (health facilities (MeSH) OR health facilities OR (health AND facilities) OR (health AND care AND facilities) OR health care facilities) AND (survey OR data collection (MeSH) OR data collection OR (data AND collection)) AND (electricity (MeSH) OR electricity). We also searched the Ministry of Health (MOH) and National Bureau of Statistics (NBS) websites of 46 sub-Saharan African countries. No time frame was specified in the searches. For one of the countries (Liberia), we obtained permission directly from the Ministry of Health and Social Welfare to review national data being analyzed in the context of a donor-supported study on electrification and maternal and newborn service delivery.^14^

### Data Inclusion Criteria

For inclusion, a study and/or the data set had to meet all of the following criteria:

1. Collected in or after 2000
2. Nationally representative of a sub-Saharan African country
3. Covers both public and private health care facilities
4. Includes a clear definition of “access to electricity” and description of how it was assessed

If a study or data set met the inclusion criteria but the raw data were not publicly available, we attempted to contact the corresponding author or surveying agency to obtain the data. Obtaining raw data allowed us to extract the information systematically, based on consistent assumptions and indicator definitions. In the case of Nigeria and The Gambia, where raw data were not obtained, we used reported data, insofar as clear definitions of indicators were stated and aligned with this study's variable definitions.

### Definitions and Parameters of Energy Indicators

While surveys administered by different agencies and in different countries usually contained similar types of questions, they were far from harmonized tools. Subtle but often significant inconsistencies in actual survey questions administered by different countries reflect the lack of a clear and universal set of indicators of health facilities' electricity access. For the purposes of this analysis, we therefore conducted an initial mapping of the types of survey questions most commonly posed, grouping them thematically into a typology of access issues (see supplementary Appendix Table). This close examination of individual survey questions also allowed for systematic consideration of a second critical issue: defining which survey questions, or combination of questions, from different country surveys could be directly compared in various aspects of the data analysis.

We defined 3 key electricity indicators covered in the surveys, which we analyzed in this review:

1. **Electricity Access:** A facility using, at least *some* of the time, any source of electrical power (yes or no). Facilities that reported a generator as their only source of electricity were classified as not having access to electricity if they reported that the generator was not functioning.
2. **Source of Electricity:** (1) Generator only, or (2) central supply, solar, or other source.
3. **Reliable Electricity:** Power available during all regular service hours, with no outages exceeding 2 hours on a given day in the week prior to data collection.

We disaggregate this data for 2 key categories of health facilities: “hospitals” providing tertiary care and “all other” facilities. Further disaggregation was confounded by the lack of clear definitions across countries of “primary” and “second-tier” facilities.

### Statistical Analysis

When raw data sets were available, we used the statistical software Stata (version 12) to extract country-level estimates for electricity access, source of electricity, and reliability of electricity. We applied sample weights, when available, to derive summary descriptive statistics (means) for individual countries. We derived 2 sets of estimates for each country data set: one at an aggregate level, which included all facilities in a country, and the other facility type (“hospital” and “other facilities”).

In cases where an overall mean value for a given variable (for example, access to electricity) for a type of facility is presented in our analysis, it was a simple average of the individual country averages for that variable in that facility type; we did not apply further weighting methods (for example, weighting by population). In cases where multiple surveys were available for an individual country, we used the most recent survey to derive multi-country means for a variable.

For countries with 2 years of survey data, we conducted a limited trend analysis by dividing the change in the percentage of facilities with electricity access by the number of years between surveys. Although different facilities were likely to have been assessed in the different survey years, we presumed the change in electrification was reflective of a general national trend since the surveys involved nationally representative samples of health facilities and used the same survey tool.

## RESULTS

### Study Selection

A total of 13 health facility assessments from 11 countries met the inclusion criteria, covering a time frame of 11 years (2001 to 2012). For 2 countries, we identified studies from 2 different years: Kenya (2004, 2010)^15^-^16^ and Rwanda (2001, 2007).^17^-^18^ In these cases, we used the most recent data for most aspects of the analysis but considered data from both years to analyze trends.

We identified the included data sets as follows: The PubMed literature search returned 115 publications; we reviewed every abstract in its entirety and fully reviewed 10 publications. Of these, 3 publications met the inclusion criteria and were included in our analysis (Ethiopia, The Gambia, and Nigeria).^19^-^21^ The studies for Ethiopia and Nigeria focused specifically on electricity access for facilities providing emergency obstetric and newborn care services. We also obtained nationally representative data for another 8 countries (Ghana, Kenya, Namibia, Rwanda, Sierra Leone, Tanzania, Uganda, and Zambia) by searching dedicated websites, including the SPA and SARA.^15^,^17^,^22^-^27^ Our search of the sub-Saharan African NBS and MOH websites failed to identify any data meeting the inclusion criteria. Four NBS websites and one MOH website were not functioning or could not be accessed due to security restrictions. The Liberian Ministry of Health data included surveys of virtually all public facilities for the years 2011 and 2012 but not of private facilities; as a result, we consider the data in a case study.^28^-^29^

The Technical Evaluation surveys from the Global Fund and the SAM surveys from WHO included data on electricity access, but they were not nationally representative and thus were excluded from our analysis. In addition, certain data collection methods in these 2 surveys were less consistent or robust than in other surveys; for example, the 2 surveys relied on district officers to report the percentage of facilities with electricity access compared with onsite interviewing methods used by other surveys.

### Study Characteristics

All 11 country surveys provided comparable data on *electricity access*. Survey data from 9 countries reported the *source of electricity*. In 4 of the countries (Kenya, Namibia, Sierra Leone, and Uganda), survey questions more clearly and reliably articulated the choices of solar sources used alone or in combination with other sources. For these 4 countries, we performed limited sub-analyses to explore the use of solar power in health facilities. A total of 8 surveys yielded data on *reliability of electricity supply* (Table 2). Some surveys did not specify a time frame for electricity outages while others asked about availability of electricity on the day of the survey.

**Table 2.** Electricity Access for Health Care Facilities in Selected sub-Saharan African Countries, by Facility Type

	Percentage With:			
Country, Year (No. of Facilities)	No Electricity	Generator Only	Central, Solar, or Other Supply^a^	Reliable Electricity
**Ethiopia, 2008 (N = 797)**^20^				
All facilities	14	5	81	–
Hospital	1	2	96	–
Other facilities	15	6	79	–
**The Gambia, 2004 (N = 12)**^19^				
All facilities	0	33	67	25
Hospital	0	20	80	40
Other facilities	0	43	57	14
**Ghana, 2002 (N = 428)**^22^				
All facilities	31	–	–	–
Hospital	6	–	–	–
Other facilities	34	–	–	–
**Kenya, 2010 (N = 695)**^15^				
All facilities	26	2	72	15
Hospital	2	2	96	24
Other facilities	28	2	70	14
**Namibia, 2009 (N = 411)**^23^				
All facilities	4	1	94	49
Hospital	0	0	100	64
Other facilities	5	2	93	47
**Nigeria, 2011 (N = 121)**^21^				
All facilities	30	–	–	–
Hospital	0	–	–	–
Other facilities	32	–	–	–
**Rwanda, 2007 (N = 538)**^17^				
All facilities	18	6	76	41
Hospital	2	10	88	52
Other facilities	19	5	75	40
**Sierra Leone, 2011 (N = 106)**^27^				
All facilities	35	10	54	14
Hospital	0	4	96	23
Other facilities	37	10	53	14
**Tanzania, 2006 (N = 611)**^24^				
All facilities	50	2	47	19
Hospital	2	6	92	23
Other facilities	52	2	45	19
**Uganda, 2007 (N = 491)**^25^				
All facilities	58	1	41	15
Hospital	1	5	94	16
Other facilities	60	1	38	15
**Zambia, 2005 (N = 430)**^26^				
All facilities	20	1	78	46
Hospital	2	7	92	33
Other facilities	21	1	78	47

Because of rounding, the sum of the percentages in the first 3 columns (no electricity, generator only, and central, solar, or other supply) may not total 100.

a Includes facilities that reported use of a combination of multiple power sources (for example, central supply and generator).

### Electricity Access

On average, 74% of facilities had access to electricity (Table 3) (range = 42% to 100%). There were substantial differences in the degree of electricity access for hospitals compared with “other” facilities. For hospitals, 94% to 100% of facilities had access to electricity. For “other” facilities, only 72% of facilities, on average, had access to electricity across the 11 countries.

**Table 3.** Energy Access Among Health Care Facilities (Mean), by Facility Type, Selected sub-Saharan African Countries^a^

Energy Access	Facility Type		
	All Facilities	Hospitals Only	Other Facilities Besides Hospitals
**Access to electricity**, % (N = 11 countries)	74	99	72
**Source of electricity**, % (N = 9 countries)			
Generator only	7	6	8
Central, solar, or other	68	93	65
**Reliable electricity**, % of electrified facilities (N = 8 countries)	28	34	26

a Data for access to electricity are averages among 11 countries (Ethiopia, The Gambia, Ghana, Kenya, Namibia, Nigeria, Rwanda, Sierra Leone, Tanzania, Uganda, and Zambia); for source of electricity, among 9 countries (excludes Ghana and Nigeria); and for reliable electricity, among 8 countries (excludes Ethiopia, Ghana, and Nigeria).

### Source of Electricity

The proportion of facilities relying on only a generator for electricity ranged from an average of only 1% of facilities in Uganda and Zambia to 33% in The Gambia, yielding a mean of 7% across 9 countries. By facility type, an average of 6% of hospitals and 8% of “other” health facilities reported generators as their only source of power (Table 3). Hospitals and other facilities in The Gambia reported higher reliance on generators as their only source of power than facilities in any other country. Excluding The Gambia, 4% of all facilities, on average, relied on only generators for electricity.

In Kenya, Namibia, and Uganda, surveys asked whether a combination of central and solar sources were used. Notably, in Uganda, approximately 15% of hospitals and almost 2% of other health facilities reported using a combination of both central and solar sources. In comparison, in Kenya and Namibia, less than 3% of hospitals and less than 1% of other health facilities reported energy from a combination of solar and central supply sources.

Solar energy sources are growing in popularity. In Uganda, 15% of hospitals use both central and solar sources.

Unlike other surveys, the SARA survey of Sierra Leone asked facilities to report all sources of electricity. No distinction was made, however, between primary and secondary or backup sources, so responses add up to more than 100% for all source categories. However, results do reflect the widespread presence of solar systems, along with more conventional generator and grid sources (Table 4). Across all facilities, over one-third received some power from solar, over one-quarter from a generator, and over one-tenth from a central grid supply. Almost all hospitals reported using a generator for power, and just over half reported having a central supply.

**Table 4.** Source of Electricity for Health Care Facilities, by Type of Facility, Sierra Leone, 2012

	Facility Type		
Electricity Source	All Facilities	Hospitals Only	Other Facilities Besides Hospitals
Central grid, %	13	58	12
Generator, %	25	95	22
Solar system, %	36	43	36
Other,^a^ %	15	21	15

The total sum of sources for a particular type of facility do not add up to 100% because each facility could report more than one electricity source.

a Flashlights were the most typical response for “other” sources of electricity, reflecting a blurring of the lines between actual electricity sources and specific devices, which needs refinement in future surveys.

### Reliability of Supply

On average, only 28% of all facilities with electricity access reported reliable access (Table 3) (range = 15% to 49%). Among hospitals, 34%, on average, reported reliable electricity access (range = 16% to 64%) compared with 26% for other health facilities (range = 14% to 47%). A sub-analysis of 6 countries where generator functionality was assessed by SPA surveys found that, among facilities with generators, a low proportion (10% to 29%) reported having functional generators with fuel available at the time of the assessment.

Only 34% of hospitals in sub-Saharan Africa, on average, have reliable electricity access.

### Trends in Countries Over Time

On average, electricity access increased annually by 1.5% in Kenya and by 4% in Rwanda in the years between the two studies (Table 5). At the end of the periods studied, a much higher proportion of hospital facilities continued to have electricity access than the proportion of “other” facilities. However, those “other,” non-hospital facilities had made greater progress, on average, in providing electricity access.

**Table 5.** Trends in Electricity Access in Health Care Facilities, by Facility Type, Kenya and Rwanda

	All Facilities		Hospitals Only		Other Facilities Besides Hospitals	
Country and Year	Percentage	Annual Percentage Change	Percentage	Annual Percentage Change	Percentage	Annual Percentage Change
**Kenya**						
2004	65	1.5	98	0	63	1.5
2010	74		98		72	
**Rwanda**						
2001	58	4	92	1	52	5
2007	82		98		81	

In Kenya, electricity access increased annually in other facilities by 1.5%, and in Rwanda by 5% annually, compared with 0% and 1%, respectively, in hospitals.

## DISCUSSION

This analysis frames some of the key issues and challenges faced in defining and measuring electricity access in health facilities in developing countries. It also provides initial baseline data on electricity access in 11 sub-Saharan African countries. On average, over one-quarter of all health care facilities lacked any access to electricity, and close to three-quarters lacked access to a reliable supply of electricity. Although these data represent only about one-quarter of sub-Saharan African nations, they include 6 of the 10 most populous countries (Nigeria, Ethiopia, Tanzania, Kenya, Uganda, Ghana).

These findings reflect the significant energy insecurity not only at the primary care level but also in hospitals—the highest tiers of health care provision. These results also reveal the important role that generators have in powering health facilities in some sub-Saharan African countries, while at the same time reflecting their unreliability, evident in the high proportion of facilities reporting generators not functioning or lacking fuel.

In sub-Saharan Africa, even the highest tiers of health care—hospitals—have significant energy insecurity.

On the positive side, the results of the limited time-trend analysis illustrate that rapid progress can be achieved in improving the energy access situation in health care facilities. For example, in Rwanda, where the change was most marked, the percentage of all facilities with access to electricity rose from 52% in 2001 to 82% in 2007. Data from countries such as Liberia, Sierra Leone, and Uganda also illustrate the growing importance of solar electricity sources in health facilities, both as a stand-alone source and in combination with generators or grid supply.

### Better Measurement Needed to Inform Energy Policymaking

Analysis of these data are an initial, but significant, step in proposing parameters for defining key electricity access indicators for health facilities, which may be useful in identifying gaps and monitoring trends. This is a timely endeavor in the context of both the UN SE4All initiative and the parallel aspiration of universal health coverage.

WHO and its partners already are initiating moves to improve methods for tracking electricity access, its reliability, and the growing diversity of energy sources. An initial step has been to refine and slightly expand the electricity questions in the SARA to capture a broader spectrum of the available primary and secondary electricity sources and to better measure power reliability and capacity. WHO is currently piloting this revised survey section in several countries.

This review also has highlighted the need for a broader interagency effort to advance a framework to measure uniformly and fully the diverse dimensions of sustainable energy access in health facilities. Key institutions managing facility surveys, as well as Ministries of Health and energy experts, need to work together to identify and harmonize the best survey questions and electricity indicators relevant to actual delivery of health services. Such a framework could contribute to the development of more comprehensive, routine, global energy assessments of health care facilities by WHO and its partners, as well as by national ministries of health, to support joint health sector and SE4All monitoring and reporting of energy access in health facilities. In April 2012, the SE4All Initiative announced a new “high-impact opportunity” related to energy and women's health, which focuses additional attention on the urgent need to improve electricity access to medical clinics; such political momentum may help push forward the technical initiatives.^30^

#### Survey Coverage

Broader geographic coverage of electricity access data also is clearly needed to obtain a global picture of electricity access in health care settings. Similarly, disaggregation of data by urban and rural communities and by socioeconomic setting would help identify areas of greatest need and the most vulnerable populations.

#### Variables of Interest

Since facilities in some of the surveyed countries rely on off-grid energy sources, better assessment by energy source and by combinations of sources is critical to forecasting needs and identifying optimal energy solutions in diverse settings. Current survey questions about reliability of electricity service “during normal business hours” are inherently self-limiting because health facilities are more likely to close at night if they do not have access to electricity. Indicators capturing the duration of electricity supply during evening hours or throughout the day and night may be important to the extent that electricity access may also be an enabler of nighttime services, emergency services, or longer service hours generally.^16^,^31^-^32^ Finally, since the power demands of different facility types can vary considerably, certain other power attributes of the supply, such as capacity in terms of total watt hours or kilowatt hours available daily, could be another variable needing better assessment.

### Changing Landscape of Electricity Source

The cost of renewable technologies has declined sharply in the past decade.^33^ At the same time, average global oil prices have increased, making fossil fuels for off-grid health clinics increasingly expensive and difficult to access,^33^ as reflected in the low proportion of functional generators in surveyed facilities. Small solar power facilities are becoming more affordable, and their costs can be lower than that of fossil fuel generators over time. These factors are paving the way for small-scale solar applications suited to highly resource-constrained settings.^34^-^35^

The Liberia case study reflects this changing landscape (Box). In 2012, more public primary health clinics were using solar power systems (146) than fossil fuel generators (116). About 4 clinics in every 5 that relied on solar power as their primary energy source reported having electricity available on the day of the survey compared with only about half of the clinics that relied on diesel generators as their primary electricity source.

**BOX. Liberia Case Study: Improved Electricity Access and Reliability Through Off-Grid Power Sources**

Since 2011, the Liberian Ministry of Health and Social Welfare has conducted regular infrastructure surveys, which include electricity indicators, for all government health care facilities. The data sets, covering all public facilities, are some of the most recent considered in this study, and are available for 2 consecutive years (N = 376 facilities in 2011; N =  381 in 2012).^28^-^29^ However, since approximately 200 private facilities were not included, we excluded the Liberia data sets from our larger 11-country analysis.

Still, this case study is relevant for 3 reasons:

- Public facilities may be more representative of health care access by the broader population. Some country-based reviews also suggest that public facilities may have less electricity access or use than private counterparts, making them worthy of separate consideration.^8^
- The Liberia surveys covered both primary and backup electricity sources with 6 distinct response options: community-shared, generator, solar power, other, none, lamp/torch. (For the purpose of our analysis, we considered “lamp/torch” as “none.”) Responses also could be categorized by 3 levels of service provision: first-level health clinics, health centers, and hospitals.
- Both 2011 and 2012 surveys included a question on the reliability of electricity access, although the question was posed slightly differently each year. In 2011, the survey asked, “Is electricity available during all required operational hours?” In 2012, it asked, “On the day of assessment, was electricity available at the facility?”

This permitted some initial analysis of electricity access relative to different off-grid electricity sources (generators, solar)—an issue relevant to many parts of Africa, and particularly to Liberia, where off-grid electricity is the norm.^36^

The proportion of Liberian facilities reporting electricity access of some kind increased from 54% in 2011 to 62% in 2012. Most of this increase was due to acquisition of either generators or photovoltaic (PV) solar systems. At the health clinic level, there were more facilities reporting solar systems as their primary electricity source (146) than facilities reporting generators (116) in 2012. However, generators remained more common among second-tier health centers. In 2012, a handful of facilities had connected to a community/shared source (often as a backup source), although grid connections remained rare. All hospitals reported generators as their primary electricity source in both years.

The data suggest a higher level of reliable electricity service for primary health clinics using solar-powered systems as their primary source than for those relying on generators (Table 6). This was irrespective of the “secondary” source of electricity, if any was available. Although there are many confounding factors that require further exploration, this finding suggests that solar power might be a more reliable electricity source than generators for remote health facilities. Further assessment is needed, however, to consider the technology-specific limiting factors (for example, fuel supply and maintenance logistics for generators as compared to, weather variability, and power capacity for small, affordable PV solar systems). For instance, in Liberia PV solar systems were reported to be more frequently used for dedicated devices and low-power applications, such as lighting and refrigeration, rather than as power for the entire facility (personal communication with Elaine Fletcher and Annette Kuesel, Data Managers, Liberia Institute for Biomedical Research, 2012).

**Table 6.** Reliability of Electricity in Electrified Facilities, by Facility Type and Primary Electricity Source, Liberia, 2011-2012

Facility Type and Primary Electricity Source	Electrified Facilities Reporting Reliable Electricity Access^a^	
	2011	2012
	n/N (%)	n/N (%)
**Hospital**		
Generator	16/18 (89%)	118/20 (90%)
Community/ shared	1/1 (100%)	1/1(100%)
**Health center**		
Generator	13/14 (93%)	13/20 (65%)
Solar	10/14 (71%)	5/6 (83%)
Community/shared	–	1/1 (100%)
**Health clinic**		
Generator	59/100 (59%)	61/116 (52%)
Solar	99/109 (91%)	119/146 (81%)
Community/shared	1/1 (100%)	3/3 (100%)

Data are among all public health facilities but not private facilities.

a The 2011 survey defined reliability by whether electricity was available “during all required operational hours,” whereas the 2012 survey asked whether electricity was available “on the day of the survey.”

The Liberia MoHSW surveys show, however, that it is feasible to conduct routine, national-level collection of data on electricity access, and offers models of survey questions that can help inform more robust data collection tools, including tools that are more sensitive to alternative energy sources. Capturing the full range of energy technologies now being used in terms of how well they function, how much power they generate, and for what purposes each technology is best suited can inform policies for improving health services through energy access. Such analysis can highlight the comparative advantages of different energy choices in diverse settings and identify barriers to scale up of clean, renewable energy options.

### Study Limitations

Discrepancies in survey questions created barriers to consistent multi-country analysis, accounting for variation in the number of countries covered in elements of the sub-analysis. For the 2 countries where we assessed trends over time, questionnaire inconsistencies for different years limited our analysis to a single indicator (electricity access).

Assessment of electricity access at different levels of health facilities was limited by the lack of comparable definitions for primary, secondary, and tertiary facilities. Facility classification names were inconsistent and types of care at different levels were not clearly defined in terms of the facility categories used by individual countries. For example, some surveys referred to health centers, health posts, and dispensaries, whereas others referred to Health Center I, Health Center II, and so forth. Rather than aiming for complete uniformity, future survey models should consider ways to classify the different types of facilities in any given country by 3 key service tiers (primary, secondary, and tertiary), so that multicountry data may be analyzed and reported accordingly.

Another limitation was in facility sampling methods; while nationally representative of different facility categories, methods used to select the sample were not uniform. In some countries, sampling focused only on those facilities offering “priority” interventions while others sampled all health facilities.

## CONCLUSIONS

As far as the authors can determine, this is the first multicountry analysis of electricity access in health facilities presented in the peer-reviewed literature. The data reflect a reality described anecdotally by health care workers as operating, quite literally, “in the dark,” forced to rely on the most minimal sources of light such as flashlights or polluting and dangerous kerosene lamps.^37^ Although we have not estimated the impact in terms of health, disability, and loss of life, we presume the impact is significant. Further stratification of electricity access data by urban-rural areas and by socioeconomic setting would be useful, but in light of other health service inequities, as well as anecdotal evidence, it is likely that poor and vulnerable groups suffer the most from lack of access to electricity.^8^

Access to more reliable, cleaner, and more sustainable energy sources is increasingly important in light of these realities as well as other economic, environmental, and climate realities. There is thus an urgent need to improve the geographic coverage, quality, and frequency of data collection on energy access in health care facilities.

With a more comprehensive and standardized tracking system, countries will be able to monitor progress toward powering health facilities and its impacts on health and development, forecast future energy needs, better allocate limited resources, and share experiences with new and innovative energy solutions.
